# Treatment robustness of total body irradiation with volumetric modulated arc therapy

**DOI:** 10.1016/j.phro.2024.100537

**Published:** 2024-01-13

**Authors:** Enrica Seravalli, Mirjam Willemsen-Bosman, Annelies Zoetelief, Sanne Roosenboom, Tessa Harderwijk, Lean Krikke, Gijsbert Bol, Alexis Kotte, Eline Huijboom, Karel van Loon, Bianca Hoeben

**Affiliations:** University Medical Center Utrecht, Department of Radiation Oncology, Utrecht, The Netherlands

**Keywords:** Total body irradiation, TBI, VMAT, Conformal radiotherapy techniques, Patient position verification, Junction, Robustness

## Abstract

This study evaluated the robustness of multi-isocenter Volumetric Modulated Arc Therapy Total Body Irradiation dose distribution in the overlapping region between the head-first and feet-first computed tomography scans, considering the longitudinal isocenter shifts recorded during treatment delivery. For 15 out of 22 patients, the dose distribution in the overlapping region fulfilled all three the robustness criteria. The overlapping region dose distribution of the remaining 7 cases fulfilled two robustness criteria. The dose distribution was found to be robust against daily recorded longitudinal isocenter shifts, as a consequence of the patient position verification procedure, of up to 16 mm.

## Highlights

• Volumetric modulated arc therapy total body irradiation was robust against daily recorded isocenter shifts up to 16 mm.
• For 15 patients, the head first and feet first overlapping region dose distribution met the robustness criteria.
• For 7 patients, at least two robustness criteria were met even when omitting the  feet first online correction.

## 1 Introduction

Multi-isocenter Volumetric Modulated Arc Therapy (VMAT) for Total Body Irradiation (TBI) has been introduced in various centers [1], [2], [3], [4], [5]. Early adopters describe their techniques as single-center efforts [2], [3], [6], with a variety of implementation solutions and patient position verification (PPV) imaging workflows [7]. VMAT-TBI delivery is sensitive to patient positioning errors due to the dose matching of multiple isocenters [8] and arcs simulated on different patient orientation (Head First Supine (HFS) and Feet First Supine (FFS) position) computed tomography (CT) scans [9]. The robustness of the dose distribution depends on the way the treatment planning system (TPS) allows feathering of the dose between neighboring arcs in the overlapping region of the upper and lower body treatment plans. The PPV procedure can also influence delivery of the planned dose distribution, potentially leading to over- or underdosage especially in the HFS-FFS overlap zone. Mancosu demonstrated dosimetric junction robustness for in-silico shifts of up to 10 mm [9]. However, in-vivo robustness against daily recorded isocenter shifts was not addressed in previous publications on VMAT-TBI [2], [3], [5], [10]. The aim of this study was to analyze online-corrected longitudinal HFS/FFS isocenter shifts and to evaluate the robustness of VMAT-TBI dose distribution in the HFS-FFS overlap region during treatment delivery.

## 2 Methods and materials

### 2.1 Patients

The first 22 patients (age: 5–32 years; height: 111–200 cm) treated by VMAT-TBI on an Elekta Axesse linear accelerator, equipped with an Agility multileaf collimator (Elekta AB, Stockholm, Sweden), between August 2021 and July 2023 were included. Relevant patient details are reported in the supplementary material. The retrospective analysis was approved by the local ethics committee. (Institutional Review Board approval number: WAG/mb/500028).

### 2.2 VMAT-TBI technique

A rotatable table top (Innovative Technologie Völp, Innsbruck, Austria) [2] allowed rotation from HFS to FFS orientation, while the patient remained immobilized in a whole body vacuum mattress (Renfu Medical Equipment Co, Guangzhou, China) and an open face mask (Civco Medical Solutions, Kalona, Iowa, USA). Two planning CT scans (4-mm slice thickness) were acquired: one in HFS from vertex to lower thigh, and one in FFS from toes to upper pelvis. The overlap, ∼20 cm longitudinal, between the two CT scans was used for image registration. Planning target volume (PTV) consisted of the whole body minus 5 mm distance from the body surface and excluding the lenses, kidneys and lungs as organs-at-risk. The HFS and FFS scans were registered in Monaco TPS (5.11.02, Elekta AB, Stockholm, Sweden) allowing only translations [11]. VMAT 6-MV full arc plans were optimized using a 5 mm grid spacing and a statistical uncertainty of 1 % per calculation. Prescription dose (PD) was 12 Gy in 6 (twice-daily) fractions with mean dose constraints for lungs < 8 Gy, kidneys < 10 Gy and lenses < 6 Gy, conform [12]. The PTV planning requirements were: 90 % PD > 95–99 %, 110 % PD < 5–10 % and 120 %PD < 1 %.

Depending on patient height, 4 to 7 isocenters with overlapping arcs were used (Fig. S1). Monaco automatically produces a broad dose transition of beams with different isocenters without the use of additional helping contours. The beams of the consecutive isocenters in HFS and FFS had an overlap region of 4 cm and of 6 cm, respectively. A 2.5 cm auto-flash margin and optimization parameters that support the creation of large segments were used. To minimize the potential risk of OAR overdosage due to daily setup deviations, the HFS-FFS overlapping region was placed in the upper leg region. The HFS treatment plan was created using the FFS plan as base dose (Fig. S2) [3]. The collimator was set to 0° for all the beams except for the thoracic isocenter, where it was 90°.

Daily PPV procedure comprised: CBCT imaging and online correction of the thoracic HFS isocenter (HFSocisoc) and knee region FFS isocenter (FFSocisoc), CBCT imaging of the pelvic HFS isocenter without correction (Fig. S1). The remaining isocenters were not imaged. The thoracic isocenter was online corrected because it contained the lungs and, depending on the patient height, the upper part of the kidneys. The pelvic area image was used to check for potential detrimental deviations after online correction of the thoracic isocenter. Due to thoracic online correction, setup deviations can be introduced at other - relatively distant - isocenters, especially in the presence of rotations. The anatomy imaged by the pelvic CBCT was evaluated with respect to the same anatomy on the planning CT, without applying any correction. If setup deviations > 10 mm anterior/posterior and left/right, >5 mm cranio/caudal were detected, the patient was repositioned and the PPV workflow started again. HFS radiation delivery followed HFS PPV and FFS radiation delivery followed FFS PPV.

### 2.3 Data analysis

Per fraction (total 132 fractions), the planned longitudinal distance between HFSocisoc and FFSocisoc was compared to the pre-fraction distance between these isocenters calculated *with* (wcFFS) and *without* (wocFFS) the applied online FFS patient position correction. Moreover, for each fraction the treatment plan was recalculated shifting all FFS isocenters to the pre-fraction HFSocisoc and FFSocisoc longitudinal positions wcFFS and wocFFS. Afterwards, the recalculated dose per fraction was rigidly accumulated for the 6 fractions and compared to the initial dose distribution in the HFS-FFS overlapping region for all the patients. For the dose comparison, a box of 20 cm^2^ cropped to the PTV was created, placed exactly in the middle of the most caudal HFS and most cranial FFS isocenter. The volumes of this box (V) receiving 90 %, 110 % and 120 % of PD were calculated for the three dose distributions (initial, wcFFS, wocFFS) per patient. A dose distribution in the overlapping region was considered robust and clinically acceptable when V90% PD > 95 %, V110% PD < 3 % and V120% PD < 1 %. The V90% PD, V110% PD and V120%PD statistical difference between the original and wcFFS, wocFFS accumulated shifted dose distribution per patient was assessed by the two-sided Wilcoxon signed-rank test using paired comparison in SPSS version 25 (IBM corporation) and significance was defined as p < 0.05.

## 3 Results

**Fig. 1:**
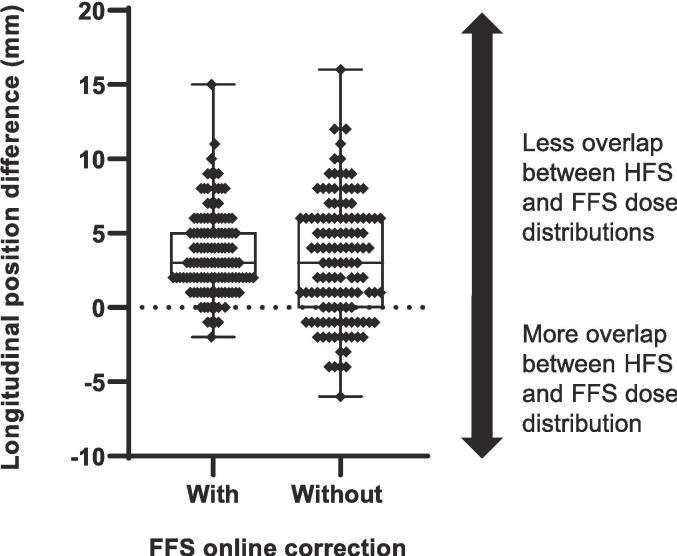
Boxplot of the difference between planned and pre-fraction CBCT-imaged longitudinal position of the HFS and FFS isocenters, with and without FFS online correction. A positive vs. negative difference corresponds to less vs. more overlap between HFS and FFS dose distributions compared to the planned situation.

The median translation found for (HFSocisoc) / [FFSocisoc] was (+1) / [0] mm lateral, (-1) / [0] mm longitudinal and (+1) / [0] mm vertical (supplementary material table S2 and table S3). The median absolute difference between the planned and the pre-fraction longitudinal position of the HFSocisoc and FFSocisoc was + 3 mm (range: 0 - 15 mm) for wcFFS, and + 4 mm (range: 0- 16 mm) for wocFFS. For 96 % of the fractions, the pre-fraction longitudinal distance between HFSocisoc and FFSocisoc showed an increase compared to the planned distance, meaning less overlap-volume of the HFS and FFS dose distributions compared to the initial situation (Fig. 1). Excluding the FFS online correction for the current analysis, showed less overlap-volume of the HFS and FFS dose distributions for 76 % of the fractions.

**Fig. 2:**
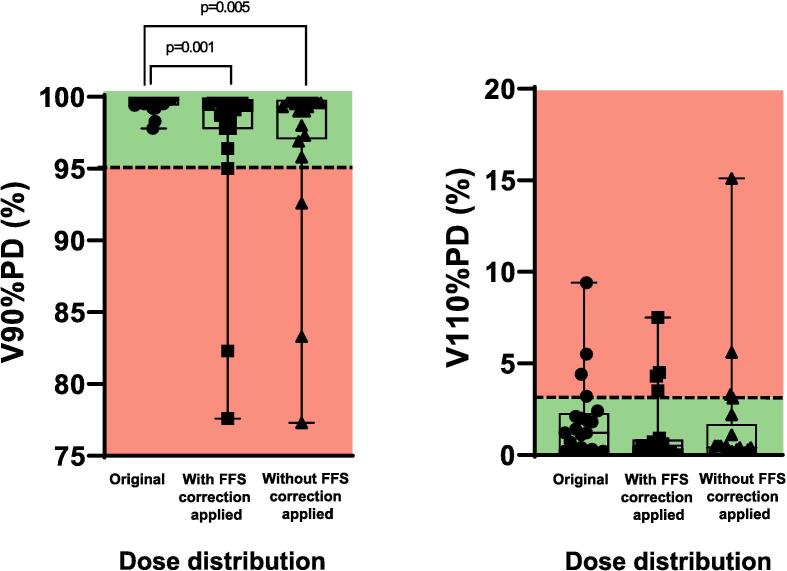
Boxplot of the volume (V) of the analyzed box in the overlapping region between HFS and FFS dose distributions receiving the 90% PD and 110% PD after in-silico reconstructed translation, with and without the pre-fraction applied FFS online correction, compared to the initially planned dose distribution in this area. The dotted line shows the acceptance criterion for robustness. Green indicates criterion fulfilled, red indicates criterion not fulfilled.

For all patients together, a significant difference was found between the V90% PD of the initial dose distribution and the wcFFS (p = 0.001) and wocFFS (p = 0.005) V90% PD, while for the V110% PD the difference was not significant (wcFFS and wocFFS both p > 0.05). The accumulated dose distribution in the analyzed overlapping region of 7/22 patients did not fulfill all three robustness criteria (Fig. 2, V120% PD not shown because the V120% PD < 1 % criterion was fulfilled for accumulated dose distributions in all patients). For 4/7 patients, the V110% PD did not fulfill the robustness criterion: for 2/4 patients both accumulated dose distributions wcFFS and wocFFS failed; for 1 patient the wcFFS dose distribution failed, and for another the wocFFS. For 3/7 patients, the V90% PD did not fulfill the robustness criterion: for 2/3 patients it concerned both wcFFS and wocFFS dose distribution while for one patient it concerned only the wocFFS dose distribution.

## 4 Discussion

For the majority (15/22) of patients, the dose distribution in the overlapping region fulfilled all three robustness criteria. For 7/22 patients, at least two of the three robustness criteria were met with or without FFS online correction.

The median absolute difference between planned and pre-fraction longitudinal position of the HFS and FFS isocenters, with and without FFS online correction applied, differed by 1 mm. However, more fractions showed a difference > 5 mm when not applying the FFS online correction. When omitting pre-fraction FFS online corrections, the dose differences in the accumulated dose distribution of the overlapping region fall mostly within the set robustness criteria, and are smaller than the inhomogeneity of a TBI dose distribution that is usually obtained by conventional techniques [13], [14], [15]. The longitudinal FFS online correction could, if needed, be safely omitted in individual patients, when i.e. an on average 5 min shorter treatment session time is desirable. At our department, it was decided to retain the FFS online correction in the PPV workflow, as an extra safety check for table position adjustment errors after patient rotation.

A systematic error was observed, where in 96 % and 76 % of the fractions, wcFFS and wocFFS respectively, the pre-fraction longitudinal distance between HFSocisoc and FFSocisoc increased compared to the planned distance, with median 3–4 mm. Next to setup error (median 1 mm), the accuracy of: the laser used to align the patient in the treatment room (0.5 mm), the treatment table calibration (0.5 mm), and the rotatable table top mechanism (1 mm) contribute to the systematic error. Moreover, reference markers on the patient are in the form of tattoos but also marked crosses. The width of the line drawn by the marker introduces a systematic uncertainty of about 1 mm. Furthermore, patients receive IV fluids in the days before TBI and the body volume of the patient can be slightly greater than at the time of planning CT. Also, some shrinkage of the mattress may occur between preparation and treatment. With the feet and shoulders fitted snugly in the mattress and stable mask-induced positioning of the head, the patient may overall adjust with relatively more compaction over the trunk area and a slight cranial push of the knees, explaining the systematic small movement – more often after FF online correction - of the thoracic isocenter away from the knee isocenter, which is placed between the legs on the mattress. Difference in patient relaxation between the planning CT and during treatment delivery may contribute to the systematic error as well.

In this study, the median translations and rotations in all directions during the online PPV procedure for both HFS and FFS treatments were 0 or 1 mm/degrees, indicating that the applied immobilization ensures a reproducible patient positioning, and that patient HF-FF rotation using the rotatable table top does not introduce extra setup uncertainty. In only 26 % of all treatment fractions, online-corrected HFS isocenter position shifts were more than 5 mm, compared to 90 % of all fractions reported by Guo et al. without use of a rotatable tabletop [6].

The primary limitation of this study is that only a limited number of patients, 22, was considered. Moreover, the accumulated dose distribution was obtained recalculating the initial dose distribution for isocenter shifts with the planning CT, not taking into account possible anatomical variations (organs at risk position or variations in body contour). However, the aim of the study was to assess the dosimetric robustness in the overlapping region located below the lower pelvis where no defined OAR was situated, and body contour changes are less likely to occur than in other regions of the body. A rigid accumulation of the fraction dose was chosen because only negligible anatomical deformations in the HFS and FFS overlapping region location were expected. The results are valid for the adopted workflow and used software and hardware, and they cannot be directly translated to other implementations of VMAT-TBI.

To conclude; the multi isocenter VMAT-TBI was found to be robust against daily recorded longitudinal isocenter shifts of up to 16 mm as a consequence of the PPV procedure. This information can be useful for departments who are considering the implementation of VMAT-TBI, and those who may want to evaluate their treatment robustness against these results.

## CRediT authorship contribution statement

**Enrica Seravalli:** Conceptualization, Methodology, Formal analysis, Writing – original draft. **Mirjam Willemsen-Bosman:** Writing – review & editing. **Annelies Zoetelief:** Writing – review & editing. **Sanne Roosenboom:** Writing – review & editing. **Tessa Harderwijk:** Writing – review & editing. **Lean Krikke:** Writing – review & editing. **Gijsbert Bol:** Conceptualization, Writing – review & editing. **Alexis Kotte:** Conceptualization, Writing – review & editing. **Eline Huijboom:** Writing – review & editing. **Karel van Loon:** Writing – review & editing. **Bianca Hoeben:** Writing – review & editing.

## Declaration of Competing Interest

The authors declare that they have no known competing financial interests or personal relationships that could have appeared to influence the work reported in this paper.
