# Supplementary material for: Treatment robustness of total body irradiation with volumetric modulated arc therapy
Source: Phys Imaging Radiat Oncol. 2024 Jan 13;29:100537. doi: 10.1016/j.phro.2024.100537 (PMC10827537; doi:10.1016/j.phro.2024.100537)
Supplement: Supplementary data 1 [file mmc1.docx]

**Supplementary material**

Table S1. Patient height, number of isocenters of the head first supine (HFS) and feet first supine (FFS) treatment plan, average dose to PTV, lungs and kidneys and homogeneity index (HI=D5%/D95% of the PTV). *Patient 8 had leukemic deposits in the kidneys so these were not spared.

| Patient | Height | Number of isocenters | | Dmean lungs | Dmean kidneys | Dmean PTV | HI-PTV |
| --- | --- | --- | --- | --- | --- | --- | --- |
|  | cm | HFS | FFS | Gy | Gy | Gy |  |
| 1 | 191 | 4 | 3 | 5.6 | 3.5 | 12.2 | 1.2 |
| 2 | 173 | 3 | 3 | 7.8 | 7.1 | 12.3 | 1.2 |
| 3 | 111 | 3 | 1 | 6.9 | 9.2 | 12.6 | 1.1 |
| 4 | 166 | 4 | 3 | 6.0 | 6.3 | 12.1 | 1.1 |
| 5 | 119 | 3 | 2 | 5.8 | 7.4 | 12.2 | 1.1 |
| 6 | 200 | 3 | 3 | 7.9 | 8.2 | 12.2 | 1.2 |
| 7 | 167 | 3 | 3 | 6.6 | 3.7 | 12.1 | 1.1 |
| 8* | 190 | 4 | 3 | 7.6 | 12.0 | 12.2 | 1.1 |
| 9 | 166 | 3 | 3 | 7.1 | 6.7 | 12.0 | 1.2 |
| 10 | 166 | 3 | 3 | 5.6 | 7.0 | 12.4 | 1.1 |
| 11 | 156 | 4 | 2 | 7.2 | 6.1 | 12.3 | 1.1 |
| 12 | 140 | 3 | 2 | 5.5 | 6.5 | 12.4 | 1.1 |
| 13 | 173 | 4 | 3 | 7.0 | 7.7 | 12.3 | 1.2 |
| 14 | 186 | 4 | 3 | 6.6 | 7.3 | 12.3 | 1.2 |
| 15 | 149 | 3 | 2 | 6.2 | 7.5 | 12.4 | 1.1 |
| 16 | 145 | 4 | 2 | 6.3 | 8.1 | 12.3 | 1.1 |
| 17 | 145 | 3 | 2 | 5.0 | 7.6 | 12.3 | 1.1 |
| 18 | 170 | 4 | 3 | 4.7 | 6.7 | 12.2 | 1.2 |
| 19 | 152 | 3 | 2 | 4.3 | 6.2 | 12.1 | 1.2 |
| 20 | 176 | 4 | 3 | 6.6 | 3.2 | 12.0 | 1.2 |
| 21 | 168 | 4 | 3 | 6.7 | 6.9 | 12.2 | 1.1 |
| 22 | 137 | 3 | 2 | 5.6 | 6.6 | 12.4 | 1.1 |
| Median | 166 | - | - | 6.0 | 7.0 | 12 | 1.0 |
| [Min ÷ Max] | 111 ÷ 200 |  |  | 4.0 ÷ 8.0 | 3.0 ÷ 12.0 | 12.0 ÷ 13.0 | 1.1 ÷ 1.2 |


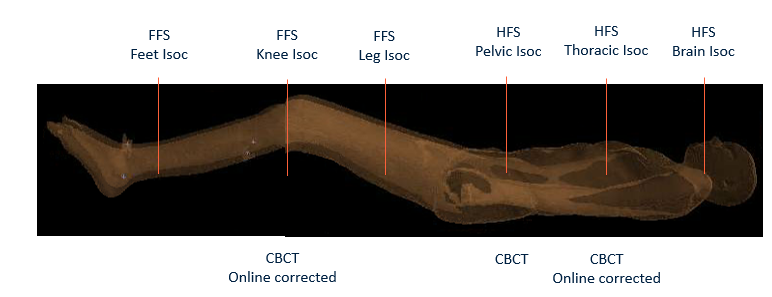


Figure S1 Example of isocenter placing for a 156 cm tall patient


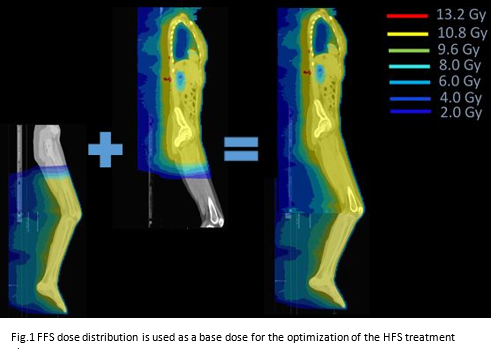


Figure S2 The Feet First Supine dose distribution is used as a base dose for the optimization of the Head First Supine treatment plan

Table S2 Translations and rotations for the CBCT-imaged and online corrected HFS isocenter

|  | Right/left  (mm) | Cranio/caudal  (mm) | Anterior/posterior  (mm) | Pitch (°) | Roll (°) | Yaw (°) |
| --- | --- | --- | --- | --- | --- | --- |
| Median | +1 | -1 | +1 | 0 | 0 | 0 |
| Min | -11 | -7 | -10 | -1 | -2 | -3 |
| Max | +7 | +9 | +13 | +2 | +4 | +3 |

Figure S3 Translations and rotations for the CBCT-imaged and online corrected HFS isocenter

Table S3 Translations and rotations for the CBCT-imaged and online corrected FFS isocenter

|  | Right/left  (mm) | Cranio/caudal  (mm) | Anterior/posterior  (mm) | Pitch (°) | Roll (°) | Yaw (°) |
| --- | --- | --- | --- | --- | --- | --- |
| Median | 0 | 0 | 0 | 0 | 0 | 0 |
| Min | -9 | -6 | -8 | -3 | -3 | -2 |
| Max | +7 | +4 | +9 | +3 | +4 | +1 |

Figure S4 Boxplot of Translations and rotations for the CBCT-imaged and online corrected FFS isocenter
